# Expression of Recombinant Human Octamer-Binding Transcription Factor 4 in Rice Suspension Cells

**DOI:** 10.3390/ijms22031409

**Published:** 2021-01-30

**Authors:** Li-Fen Huang, Desyanti Saulina Sinaga, Chia-Chun Tan, Shu-Ju Micky Hsieh, Chi-Hung Huang

**Affiliations:** 1 Graduate School of Biotechnology and Bioengineering, Yuan Ze University, 135 Yuan-Tung Road, Zhongli, Taoyuan County 320, Taiwan; desyanti@g.ncu.edu.tw (D.S.S.); smallbu1023@yahoo.com.tw (C.-C.T.); 2 Taiwan Advance Bio-Pharmaceutical Inc., 12F, No. 25, Ln. 169, Kangning St, Xizhi Dist., New Taipei City 221, Taiwan; mickyhsieh@biotest.com.tw (S.-J.M.H.); chh@biotest.com.tw (C.-H.H.)

**Keywords:** *αAmy3* promoter, recombinant human embryonic transcription factor, human Oct4, rice cell suspension culture system

## Abstract

The rice cell suspension culture system is a good way to produce recombinant human proteins, owing to its high biosafety and low production cost. Human Octamer-binding Transcription Factor 4 (Oct4) is a fundamental transcription factor responsible for maintaining human pluripotent embryonic stem cells. Recombinant Oct4 protein has been used to induce pluripotent stem cells. In this study, recombinant Oct4 proteins are produced via a sugar starvation-inducible *αAmy3*/*RAmy3D* promoter–signal peptide-based rice recombinant protein expression system. *Oct4* mRNAs accumulate in the transgenic rice suspension cells under sugar starvation. The Oct4 recombinant protein is detected in the transgenic rice suspension cells, and its highest yield is approximately 0.41% of total cellular soluble proteins after one day of sugar starvation. The rice cell-synthesized recombinant human Oct4 protein show DNA-binding activity in vitro, which implies that the protein structure is correct for enabling specific binding to the target DNA motif.

## 1. Introduction

There has been a recent surge in applications of human-induced pluripotent stem cells (iPSCs) in therapeutics research, such as in drug screening, disease modeling, and gene identification, together with the potential for patient-specific tissue replacement [1,2,3]. Human Octamer-binding Transcription Factor 4 (Oct4) is a homeodomain transcription factor that belongs to the POU (Pit-Oct-Unc) family and acts as a pioneer factor to initiate reprogramming of fibroblasts into iPSCs [4]. Oct4 contains a DNA-binding domain and activates target genes by recognizing the consensus sequence ATGCAAAT in promoter or enhancer regions. Oct4 is mainly expressed in unfertilized oocytes, zygotes, early embryos, and primordial germ cells [5,6], and functions together with SOX2 and NANOG to regulate self-renewal and pluripotency of embryonic stem cells [7]. In addition, in vitro cell reprogramming studies indicate that *Oct4* is expressed abundantly in embryonal carcinoma cells and embryonic stem cells [8,9]. Ectopic expression of *Oct4* and other reprogramming factors can reprogram somatic cells into iPSCs [10]. The virus-mediated gene transfer process is a common method to deliver these reprogramming factor’s genes. However, the approach may result in unwanted genomic mutations, residual expression, and reactivation of transgenes [11]. Therefore, several transgene-free approaches were developed to avoid or eliminate the integration of transgenes in the reprogrammed cells [12,13,14,15,16]. Direct transduction of reprogramming factor proteins is one of the transgene-free approaches, which adds recombinant reprogramming factor proteins, such as the Oct4, to the culture medium of host cells to induce reprogramming [17].

To derive the recombinant Oct4 for iPSCs formation, several protein expression platforms have expressed recombinant human Oct4, such as mammalian cells [18], insect cells [19], the yeast *Pichia pastoris* [20], and *Escherichia coli* [21], but some problems have been encountered. In mammalian expression systems, recombinant Oct4 proteins are limited by low yields, cumbersome manipulations, and high culture costs [18]. In baculovirus-infected Sf9 insect cells, the secreted recombinant Oct4 proteins were detectable only in cell debris and not in the cell culture medium [19]. Production of Oct4 in *P. pastoris* is strongly induced by methanol [20], but there are concerns about the toxic and inflammatory nature of methanol. In *E. coli*, recombinant Oct4 proteins are aggregated at inclusion bodies, hence additional denaturation and recovery steps are required during protein purification [21].

A plant cell system is a promising recombinant protein production platform owing to the capacity for post-translational modification, as well as the low production cost. In addition, a plant cell system faces little or no risk of human pathogen contamination, and therefore, offers a high level of biosafety compared with current commercial mammalian and microbial host cells [22]. Rice is a low-allergen staple food and a model research plant. With advantages, such as well-developed genetic transformation technology [23,24], short cell-doubling time, and straightforward downstream protein purification, a rice cell suspension culture system is recognized as an excellent host cell for recombinant protein production [25]. Several recombinant proteins, such as human serum albumin [26], cytokines [27,28,29], antibodies, and vaccines [30,31], have been produced successfully by a rice cell suspension culture system, which produces competitive yields of several recombinant proteins among several plant species [32].

The most widely applied transgenic rice cell suspension culture system is based on the rice *α*-amylase promoter *αAmy3* (also termed *RAmy3D*), which is a sugar starvation-inducible promoter [33], and its signal peptide [34]. In the present study, we constructed and transformed the human *Oct4* gene controlled by the *αAmy3* promoter and its signal peptide into rice suspension cells [35]. Several independent transgenic suspension cell lines were obtained. Expression of *Oct4* mRNAs and recombinant human Oct4 was monitored in these transgenic suspension cell lines in sugar-supplemented and sugar-starved media. In addition, the DNA-binding ability of the rice-derived recombinant human Oct4 protein was compared with that of commercial recombinant TAT-Oct4, an Oct4 fusion protein carrying the cell-penetrating the TAT domain from HIV.

## 2. Results and Discussion

### 2.1. Generation of Transgenic Rice Cell Lines Harboring αAmy3p-SP-Oct4 Gene

**Figure 1:**
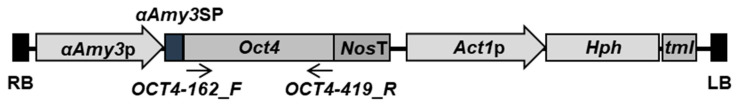
Schematic representation of the human *Oct4* expression cassette in transgenic rice plants. The human *Oct4* cDNA was inserted downstream of the *αAmy3* promoter (*αAmy3*p)–signal peptide (*αAmy*3SP) sequence. Expression of the hygromycin phosphotransferase resistance (*Hph*) gene was driven by an actin promoter (*Act1*p), and the terminator of the *Agrobacterium tml* gene was used. RB, T-DNA right border; Nos T, Nopaline synthase terminator; LB, T-DNA left border. Two primers used to amplify the inserted *Oct4* gene were designated OCT4-162_F and OCT4-419_R.

The sugar starvation-inducible *αAmy3* promoter (*αAmy3*p) and its signal peptide (*αAmy3* SP) have been used successfully to express diverse recombinant proteins in rice suspension cells [25]. To produce the recombinant human Oct4 transcription factor in rice suspension cells, full-length *Oct4* cDNA was inserted downstream of *αAmy3*p and *αAmy3*SP (Figure 1) in a Gateway-compatible T-DNA destination vector. The T-DNA expression cassette was transformed into rice cells via an *Agrobacterium*-mediated plant transformation system. Several stable transgenic rice calli were obtained (Supplemental Figure S1A). Four independent transgenic rice calli that produced high levels of *Oct4* mRNA, namely, Oct4-a1, Oct4-5, Oct4-6, and Oct4-8 (Supplemental Figure S1B), were selected and used to establish suspension cell lines.

### 2.2. Recombinant Human Oct4 Proteins Were Produced by Transgenic Rice Suspension Cell Lines

**Figure 2:**
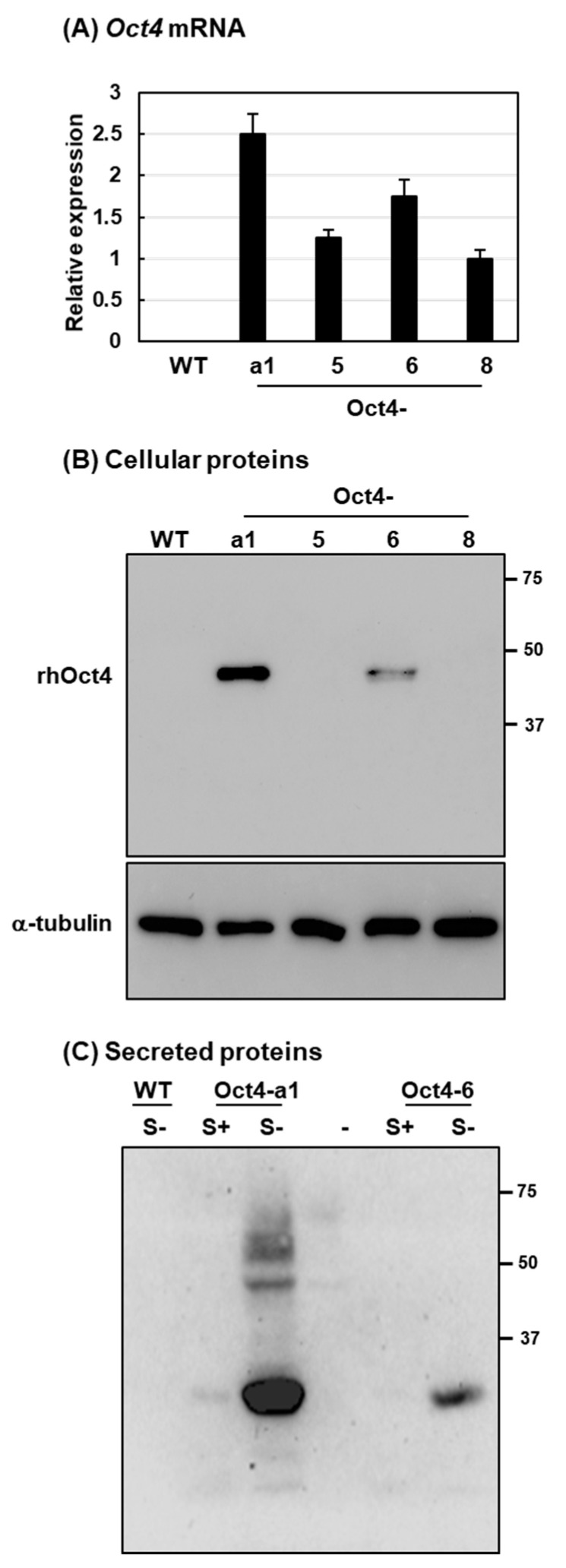
Establishment and characterization of *Oct4* transgenic suspension cell lines. (**A**) Expression of *Oct4* in rice suspension cells. Total RNA was isolated from sugar-starved cells cultured for two days and then analyzed by qRT-PCR using *Oct4*-specific primers. WT is the non-transformed wild-type line used as a negative control. Error bars indicate the standard deviation (SD) of triplicate experiments. Gene expression was relative to that of Oct4-8 cells, with 1 = equivalence. (**B**) Suspension cells of the WT and four *Oct4* transgenic lines (Oct4-a1, Oct4-5, Oct4-6, and Oct4-8) were cultured in a sugar-free MS medium for two days. Total soluble proteins were isolated to determine recombinant human Oct4 (rhOct4) abundance by western blot analysis with specific Oct4 antibodies. α-Tubulin was detected as the loading control. (**C**) WT, Oct4-a1, and Oct4-6 suspension cells were cultured in a sugar-free MS medium with 12% (v/v) cell concentrations for two days. The culture media were collected and condensed 10-fold after freeze-drying. The media were used to determine rhOct4 abundance by western blot analysis with anti-Oct4 antibodies.

To examine *Oct4* expression in the four selected rice suspension cell lines, 3% (*v*/*v*) of each sample of rice cells was incubated in sucrose-containing culture medium for three days before being transferred to sucrose-free medium for two days. To analyze expression levels of *Oct4* mRNA and the recombinant human Oct4 (rhOct4) protein, quantitative real-time PCR (qRT-PCR) and immunoblotting analysis were performed, respectively. The *Oct4* mRNAs were detected in the four suspension cell lines, and Oct4-a1 exhibited the highest level of *Oct4* mRNA among the cell lines (Figure 2A). The predicted 43 kDa rhOct4 protein was detected in cellular soluble proteins of two suspension cell lines, Oct4-a1 and Oct4-6. Using monoclonal anti-Oct4 antibodies, the abundance of rhOct4 was higher in Oct4-a1 than Oct4-6 (Figure 2B). In addition, rhOct4 protein was not directly detectable in the sucrose-free culture-medium for all four suspension cell lines. To increase the concentration of rhOct4 proteins in the cell-culture medium, 12% (*v*/*v*) of Oct4-a1 and Oct4-6 rice cells were incubated in sucrose-containing and sucrose-free media for two days. Ten-fold condensed cell-culture medium samples were prepared by freeze-drying. Then, the rhOct4 protein was detected in t cell-culture media for the Oct4-a1 and Oct4-6 lines by immunoblotting analysis (Figure 2C). The Oct4 protein contains conserved amino acid residues for O-glycosylation [36], and rhOct4 is secreted by a default pathway from the endoplasmic reticulum and Golgi apparatus where glycans may be added to the rhOct4 protein. Thus, a molecular weight higher than 43 kDa was observed for rhOct4 in the sugar-free culture medium of the Oct4-a1 line (Figure 2C). In addition, a high quantity of a small rhOct4 fragment, around 25 kDa, was detected in the sugar-free culture medium (Figure 2C). Small protein fragments of rhOct4 were detected in the sugar-free culture medium for both the Oct4-a1 and Oct4-6 cell lines. Based on the *Oct4* mRNA levels and intracellular rhOct4 protein levels in these transgenic lines, the results suggest that the secreted rhOct4 proteins might be degraded in the sugar-free cell-culture medium.

### 2.3. The rhOct4 Protein Is Unstable in Sugar-Free Culture Medium

**Figure 3:**
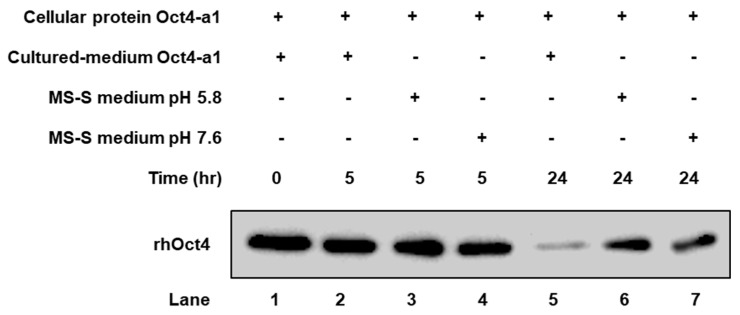
Analysis of rhOct4 protein stability in rice cell suspension culture medium. Cellular proteins extracted from Oct4-a1 suspension cells sugar-starved for two days were incubated with a cell-culture medium of Oct4-a1, fresh MS medium with pH 5.8, or fresh MS medium with pH 7.6 for 5 or 24 h. The rhOct4 abundance was determined by western blot analysis with human Oct4 antibodies.

A time-course in vitro assay of rhOct4 protein stability in the culture medium was performed to assess whether rhOct4 protein is degraded. Crude protein extracts from Oct4-a1 cells sugar-starved for two days were incubated either with a cell-culture medium or with a medium for only 5 or 24 h. Reaction mixtures were subjected to immunoblotting analysis with monoclonal anti-Oct4 antibodies. Similar abundances of rhOct4 protein were detected after 5 h incubation in the three reaction mixtures (Figure 3, Lanes 2–4). However, compared with medium-only, rhOct4 protein was only weakly detectable in the cell-culture medium after 24 h (Figure 3, Lane 5), whereas rhOct4 protein signals remained high when incubated in medium-only (Figure 3, Lanes 6 and 7). The average pH in a sugar-free cell-culture medium changed to 7.6 from the initial 5.8 during incubation for two days. To test whether the pH change affected rhOct4 protein stability, a sucrose-free Murashige and Skoog (MS) medium of pH 5.8 and 7.6 were used. The rhOct4 protein abundances were similar in the sugar-free MS medium under the two pH values (Figure 3, Lanes 6 and 7), implying that rhOct4 protein instability was not major affected by the increase of pH in a sugar-free cell-culture medium that may contain rice secretary proteases. Few recombinant biopharmaceutical proteins in a plant-based recombinant protein production system, such as monoclonal antibodies [37,38,39], have been reported in which protein fragments truncated via a proteolytic process were detected. Previous reports indicate that cysteine proteases exist in a sugar-free culture medium of rice suspension cells, and these proteases have negative impacts on the production of recombinant human granulocyte-macrophage colony-stimulating factor [40,41]. Unstable rhOct4 proteins in a sugar-free cell-culture medium might be attacked by particular proteases secreted from rice suspension cells into sugar-free medium. Our protease activity assay showed that approximately 60–70 and 150 kDa proteases were detected in sugar-deficient rice cell cultured medium (Supplementary Figure S2). By using RNAseq and proteomic analyses, several secreted proteases were found in sugar-free culture medium. If the particular proteases degrade rhOct4 protein, specific protease inhibitors can be applied to increase rhOct4 protein level in a sugar-free cell-culture medium.

### 2.4. The Highest Production of Oct4 Protein Was 0.41% of Total Soluble Proteins

**Figure 4:**
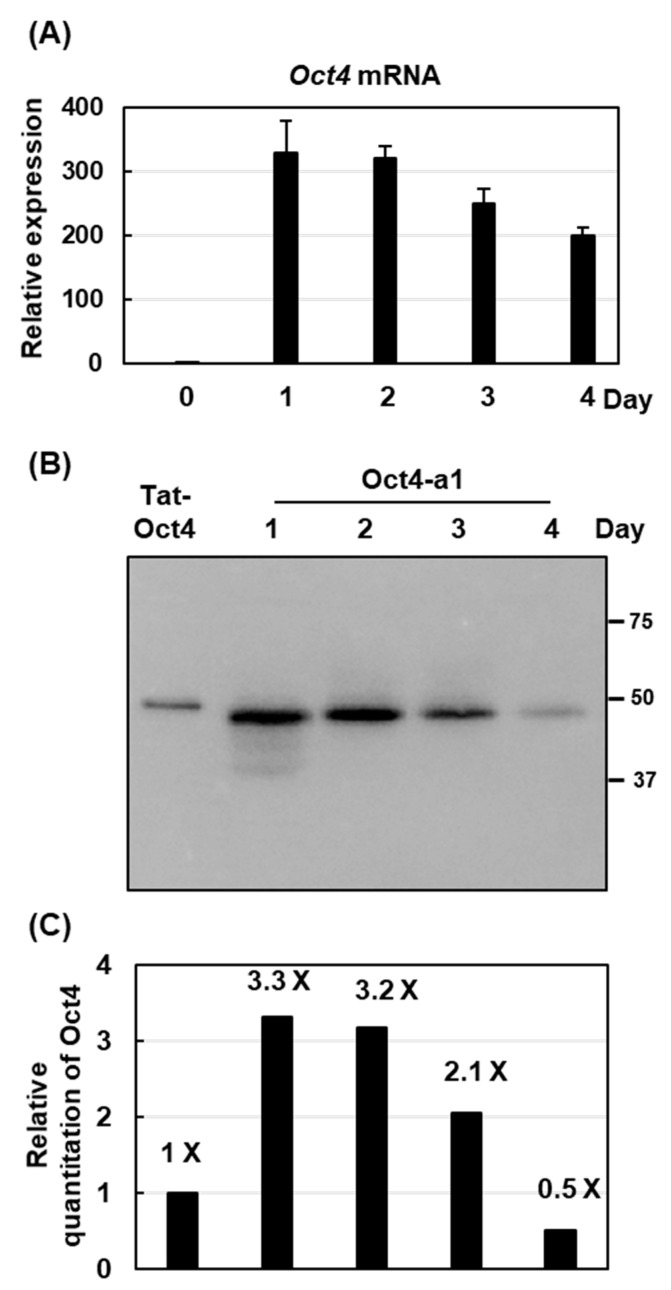
The production profile of rhOct4 in rice suspension cells. Three milliliters of Oct4-a1 suspension cells were cultured in 25 mL sugar-free MS for 1 to 4 days. Total RNA and total soluble proteins were isolated and subjected to qRT-PCR and western blot analysis. (**A**) Relative expression of *Oct4* was determined with *Oct4*-specific primers. Error bars indicate the standard deviation (SD) of triplicate experiments. Gene expression was relative to that at Day 0, with 1 = equivalence. (**B**) Western blot analysis was performed using Oct4 antibodies. Equal quantities of total protein (40 µg) per lane were loaded. Fifty grams of commercial TAT-Oct4 recombinant protein produced from *E. coli* was used as a positive control. (**C**) Relative quantification of rhOct4 protein yield in Oct4-a1 under various sugar-starvation durations was measured using a Bio-Rad Gel Doc EZ Imager system. The level of rhOct4 was relative to that of Tat-Oct4, with 1 = equivalence.

Although rhOct4 proteins were of low abundance in the suspension cell culture medium, the Oct4-a1 cell line produced a high abundance of cellular rhOct4 proteins. To determine the highest yield of cellular rhOct4 production in the Oct4-a1 suspension cell line, the Oct4-a1 cells were starved for various periods before being subjected to total mRNA and total protein extraction. The *Oct4* mRNA levels were quantified by qRT-PCR. The abundance of *Oct4* mRNA increased dramatically in cells cultured for 1 and 2 days under sugar starvation, and thereafter, gradually decreased from Day 3 to Day 4 (Figure 4A). The rhOct4 abundance during sugar-starvation periods was detected by immunoblotting analysis, and relative quantification was performed with a Bio-Rad Gel Doc EZ Imaging system using 50 ng purified TAT-Oct4 fusion protein from recombinant *E. coil* as a standard. The highest rhOct4 production was detected on Day 1 after sugar starvation (Figure 4B). The yield of rhOct4 was approximately 165 ng, which represents 0.41% of the total soluble proteins (Figure 4C).

Recombinant Oct4 proteins were produced in mammalian cells [18], insect cells [19], yeast [20], and *E. coli*. [21]. Although some reports do evaluate their protein yields, various units were used. The yield from previous studies in insect cells and yeast showed that the recombinant Oct4 yield was 6.1 mg/L [19] and 210 mg/L [21], respectively. Our present study indicates that the yield of rhOct4 was approximately 0.41% of the total soluble proteins. According to the protein levels in sugar-starved rice suspension cell, about 4534.9 μg g^−1^ cell [42], and 1 L of an initial cell density of 12% (*v*/*v*) cultured cells is roughly equivalent to 120 g of rice suspension cells, the recombinant Oct4 yield in rice cells was about 223 mg/L, implying that the productivity is now in the same order of magnitude as yeast.

### 2.5. Rice Cells Produce Biologically Active rhOct4

**Figure 5:**
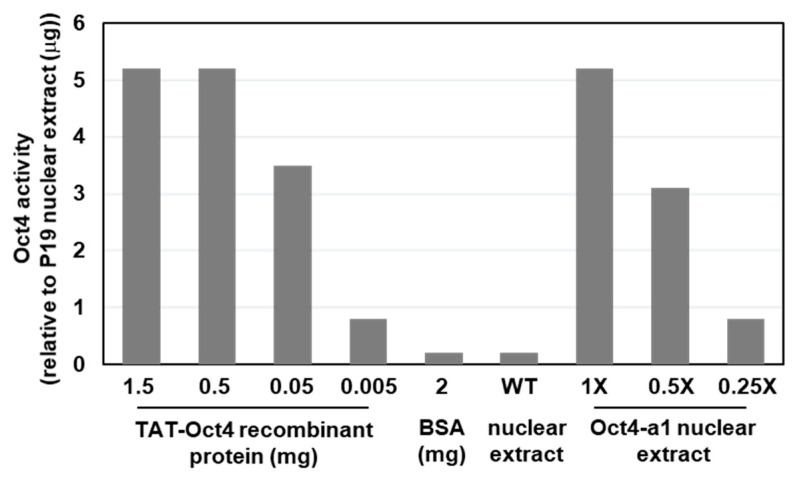
Analysis of the biological activity of rhOct4. The biological activity of rhOct4 was determined in cellular protein extract from Oct4-a1 suspension cells sugar-starved for one day. An oligonucleotide containing the Oct4 consensus binding site was incubated with three different dilutions of Oct4-a1 cellular protein extracts. Commercial TAT-Oct4 derived from *E. coli* cells was used as a reference standard, ranging from 0.005 to 1.5 mg. Two milligrams of bovine serum albumin (BSA) and wild-type rice nuclear extract (WT) were used as negative controls. The Oct4 DNA-binding activity was relative to the positive control (P19 nuclear extract).

Oct-4 belongs to the POU family and contains a bipartite DNA-binding domain consisting of the POU-specific and POU homeo-domain. The biological activity of rhOct4 was determined by its intrinsic DNA-binding ability. The DNA-binding activity of various concentrations of rice cell-derived rhOct4 and of *E. coli*-derived recombinant TAT-Oct4 was tested using the TransAM^®^ Oct-4 Transcription Factor Assay Kit. No DNA-binding activity was detected in the non-transformed wild-type line (WT) extract that contained no rhOct4 (Figure 5). In contrast, DNA-binding activity increased significantly in the presence of rice cell-derived rhOct4 in a dose-dependent manner (Figure 5). Moreover, rhOct4 activity was similar to that of *E. coli*-derived recombinant TAT-Oct4 (Figure 5). These results indicate that the rhOct4 protein was biologically active.

## 3. Materials and Methods

### 3.1. Plant Materials and Growth Conditions

Seeds of rice (*Oryza sativa* L.), Tainung 67, were dehulled, sterilized with 2.4% HClO_2_ containing Tween-20, agitated on a shaker for 30 min, washed thoroughly with sterile water, and cultured on CIM-I agar medium [23] containing 3% sucrose at 28 °C in a growth chamber under continuous light to induce callus.

To establish the rice cell suspension culture, yellow healthy embryogenic calli were transferred to MS liquid medium [43] supplemented with 3% sucrose and 10 µM dichlorophenoxyacetic acid (2,4-D) in a 125 mL flask. The cells were cultured at 28 °C on an orbital shaker at 110 rpm in a dark culture room. The suspension cells were subcultured in a fresh MS liquid medium supplemented with sucrose and 2,4-D every week.

### 3.2. Plasmid Construction

To make the *αAmy3* promoter–signal peptide–Oct4 fusion construct, the Gateway-compatible binary T-DNA destination vector, pAAmy3Dst [21], was used. The 1083-bp Oct4 cDNA fragment was amplified by RT-PCR using the forward primer (5′-CACCATGGCGGGACACCTGGCTTC-3′) and the reverse primer (5′-TCAGTTTGAATGCATGGG-3′). The fragment was inserted into the pENTR/SD/D-TOPO vector (Invitrogen) to generate the construct pENTR-Oct4, and subcloned into the pAAmy3Dst vector by LR recombination to generate the final expression vector pAAmy3-Oct4.

### 3.3. Rice Transformation

Transformation of rice was performed as described previously with a slight modification [44]. The expression vector, pAAmy3-Oct4, carrying the *αAmy3* promoter–signal peptide–Oct4 cassette was introduced into *Agrobacterium tumefaciens* strain EHA105 by electroporation. The single colony of transformed *Agrobacterium* was incubated on AB medium at 22 °C for five days. Then, embryogenic calli were incubated with the *Agrobacterium* for 20–25 min. The calli were then transferred to a co-cultivation agar medium and incubated at 22 °C in the dark for 5–7 days. The calli were rinsed 8–10 times with 250 mg/L cefotaxime in sterile distilled water, dried on sterile filter paper, transferred onto a selection medium that contained 50 mg/L hygromycin, and incubated at 28 °C. Transformed calli were regenerated on a regeneration medium supplemented with 50 mg/L hygromycin.

### 3.4. PCR-Base Genotype Analysis

Genomic DNA was isolated from each rice cell line, either from calli or suspension cells. Two micrograms of genomic DNA were subjected to PCR using a specific primer set, Oct4-162-F (5′-CTCTGAGGTGTGGGGGATT-3′) and Oct4-419-R (5′-TTGATGTCCTGGGACTCCTC-3′), to amplify the *Oct4* gene. The PCR products were separated by electrophoresis. The primers Act1-F (5′-CTGATGGACAGGTTATCACC-3′) and Act1-R (5′-CAGGTAGCAATAGGTATTACAG-3′) were used for amplification of the internal reference gene *Act1*.

### 3.5. Quantitative RT-PCR

Total RNA was isolated from rice suspension cells using the TRIzol Reagent (Sigma-Aldrich, St Louis, MO, USA). Isolated total RNA was treated with RNase-free DNase I (NEB, Ipswich, MA, USA) to eliminate possible DNA contamination. First-strand cDNA was synthesized from 2.5 µg total RNA using ReverTra Ace^®^ reverse transcriptase (Toyobo, Osaka, Japan) with oligo-dT primers. A 10-fold dilution of the resultant first-strand cDNA was subjected to qRT-PCR using the *Oct4*-specific primers Oct4-162-F and Oct4-419-R. The procedure was independently repeated at least three times. The relative gene expression was expressed as the ratio of *Oct4* mRNA abundance to *Act1* mRNA abundance. Data were analyzed using PikoReal 2.0 software (Thermo Fisher Scientific, Waltham, MA, USA).

### 3.6. Western Blot Analysis

Cells were collected from a sugar-free liquid medium after incubation at 28 °C on an orbital shaker at 110 rpm in a dark culture room. To isolate total secretory proteins from rice suspension cells, a cell-culture medium was filtered by 0.45 µm to remove cell debris. To obtain the 10-fold-concentrated cell-culture medium protein, 500 μL cell-culture medium was lyophilized in a FreeZone freeze dryer (Labconco™, Kansas, MO, USA), and the dried pellet was dissolved in 50 μL ddH2O. The concentration of protein in the supernatant was measured using a protein assay reagent (Bio-Rad, Hercules, CA, USA). Protein blot analysis was performed as described by Huang et al. [26] with an anti-human Oct4 monoclonal antibody (Merck, Darmstadt, Germany). Recombinant purified TAT-Oct4 protein from *E. coli* was used as a positive control. An ECL™ Prime Western Blotting System was used following the manufacturer’s recommended concentration to detect the protein signal with a Bio-Rad Gel Doc™ EZ imaging system.

### 3.7. Oct4 Transcription Factor Binding Assay

The Oct4 activity assay was conducted using TransAM^®^ Oct-4 Transcription Factor Assay Kits, predominantly following the manufacturer’s instructions. One gram of suspension cells was ground into fine powder in liquid nitrogen, and the fine powder was dissolved in 2 mL ice-cold lysis buffer as described in the protocol. The extracted protein sample was frozen immediately in liquid nitrogen and stored at −80 °C. Twenty microliters of sample were transferred to each well on a 96-well plate, which had been immobilized with an oligonucleotide containing the Oct4 consensus binding site (5′-ATTTGAAATGCAAAT-3′). The P19 nuclear extract provided in the kits was used as a positive control.

### 3.8. In-Gel Protease Activity Assay

Proteinase activities analysis was performed as described by Lin et al. [42]. Rice suspension cells were cultured in a sugar-free medium for two days, a cell-culture medium was filtered by 0.45 µm to remove cell debris. The culture medium was diluted 3-fold in a sample preparation buffer at 37 °C for 10 min, and then was subjected to SDS-PAGE containing 0.1% gelatin at 4 °C until the dye front reaches the bottom of the gel. The gels were then washed in 2.5% (w/v) Triton X-100 for 30 min, and incubated in potassium buffer (150 mM potassium citrate, 5 mM L-Cys, 0.1% Triton X-100, pH 5.8) for 20 h (at 37 °C). The gels were stained with Coomassie blue.

## 4. Conclusions

In this study, we developed a platform to produce recombinant human Oct4 using the *αAmy3* sugar starvation-inducible promoter and its signal peptide in a rice cell suspension culture system. The predicted 43 kDa Oct4 recombinant protein was detected among the intracellular soluble proteins and showed in vitro binding assay activity. The highest yield of Oct4 was approximately 0.41% of total soluble proteins. Three small Oct4 fragments were detected in the cell-culture medium, which implies that the secreted Oct4 proteins might be degraded by extracellular rice proteases. Therefore, future investigation of the correlation between recombinant human Oct4 protein and protease activities in the rice cell suspension culture system is recommended.

## Data Availability

The data presented in this study are available on request from the corresponding author.

## Supplementary Materials

These can be found at https://www.mdpi.com/1422-0067/22/3/1409/s1.

## Abbreviations

Oct4	Octamer-binding Transcription Factor 4
iPSCs	induced pluripotent stem cells
qRT-PCR	quantitative real-time PCR
MS medium	Murashige and Skoog medium
