# Supplementary material for: Expression of Recombinant Human Octamer-Binding Transcription Factor 4 in Rice Suspension Cells
Source: Int J Mol Sci. 2021 Jan 30;22(3):1409. doi: 10.3390/ijms22031409 (PMC7866794; doi:10.3390/ijms22031409)
Supplement: Supplementary file 1 [file ijms-22-01409-s001.pdf]

Supplemental Figure S1

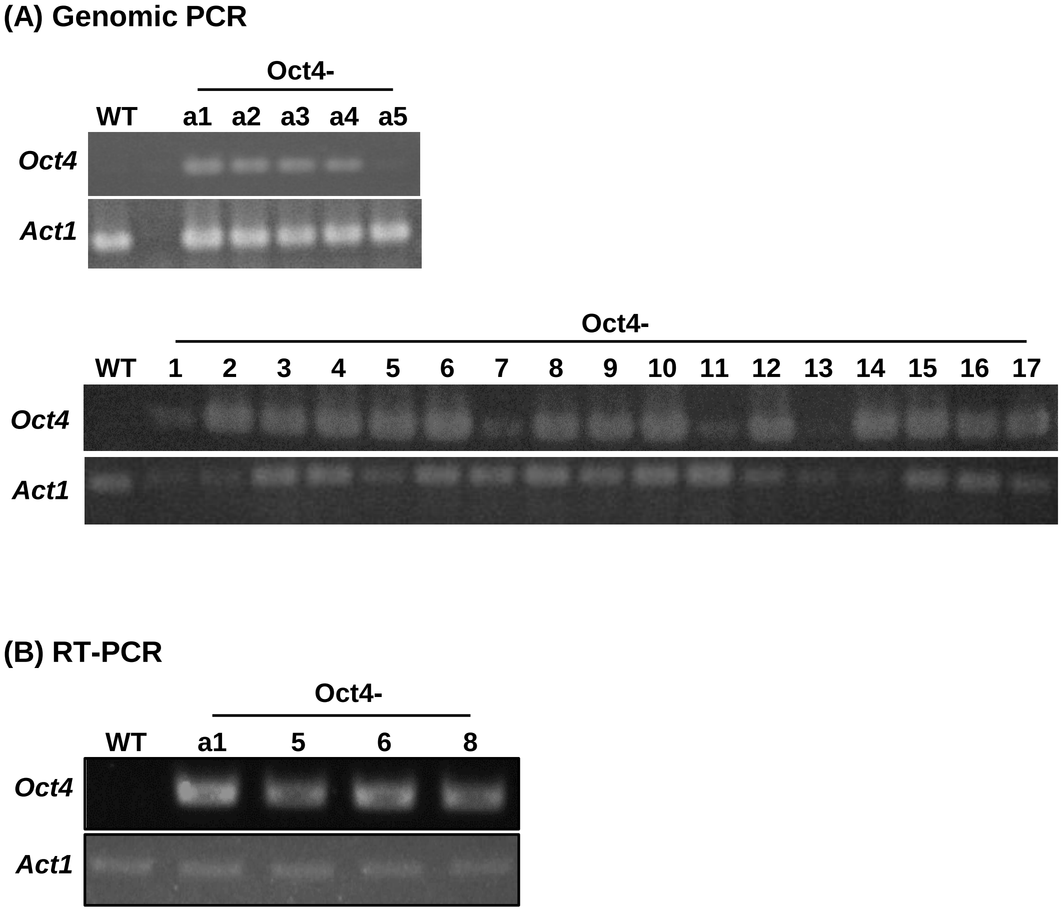

Figure S1. Characterization of *αAmy3*p- *SP-Oct4* transgenic cell lines.
A. Several independent transgenic cell lines (a1-15 and 1-17)) were obtained, and the transgene of *Oct4* were determined by PCR. Rice *Act1* DNA was used as an internal control.
B. Expression of *Oct4* in transgenic rice cell lines. Total RNA was isolated from 2 day of sugar starved cultured cells and subjected to RT-PCR using *Oct4*-specific primers. Rice *Act1* was used as an internal control. Wild-type line is indicated by WT.

Supplemental Figure S2

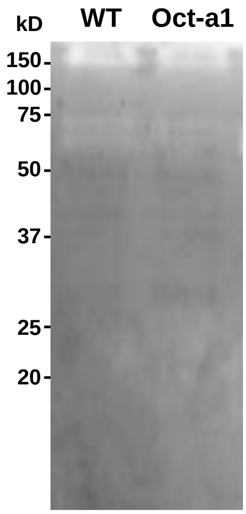

Figure S2. Examination of protease activity assay of sugar-free cultured medium. Suspension cells of the WT and Oct4-a1 lines were cultured in sugar-free MS medium for 2 days. Culture media were collected and subjected to in gel protease activity assay of sugar-free culture medium.
